# Are SEP-1 and blood culture stewardship at odds? Retrospective review of SEP-1 failures pre- and during a blood culture bottle shortage

**DOI:** 10.1017/ice.2025.10317

**Published:** 2026-02

**Authors:** Jonathan H. Ryder, Kelly A. Cawcutt, Cynthia Japp, Trevor C. Van Schooneveld

**Affiliations:** 1 Division of Infectious Diseases, Department of Internal Medicine, University of Nebraska Medical Center, Omaha, NE, USA; 2 Clinical Quality, Nebraska Medicine, Omaha, NE, USA

## Abstract

Timely blood cultures (BCx) are required by SEP-1. The recent BCx bottle shortage necessitated enhanced BCx stewardship. At two hospitals during the shortage, SEP-1 metric compliance declined related to BCx utilization. Review of cases where BCx were not obtained demonstrated most BCx were safely avoided without demonstrable patient harm.

## Introduction

In July 2024, a shortage of Becton Dickinson (BD) BACTEC blood culture (BCx) bottles required BCx stewardship efforts to mitigate the shortage.^1^ Centers for Medicare and Medicaid Services (CMS) requires reporting compliance with the Severe Sepsis and Septic Shock Early Management Bundle (SEP-1) as a pay-for-performance metric. SEP-1 requires BCx in all patients meeting sepsis criteria, and missing any bundle component results in SEP-1 failure.^2^ BCx stewardship efforts have emphasized BCx for sepsis, in part due to SEP-1.^3^ Inpatient BCx stewardship efforts have not been associated with a change in SEP-1 compliance.^4^ At our institution, BCx algorithms and guidance already existed, but stewardship efforts were enhanced during the shortage.^5^ During the BCx shortage, we observed an increased rate of SEP-1 failures related to BCx, prompting an evaluation of the relationship between the BCx shortage and SEP-1 compliance.

## Methods

A retrospective review of SEP-1 compliance data from one academic (718 beds) and one community (59 beds) hospital from 1/1/22–12/31/24 was performed and grouped by quarter. July 2024–December 2024 were designated the BCx shortage, while the preceding time was pre-shortage. Institutional BCx stewardship guidance for inpatient settings was incorporated in the electronic medical record March 2023 as part of the Johns Hopkins Prevention Epicenter Blood Culture Stewardship Collaborative.^5^ During the shortage, mitigation strategies included emphasizing local BCx guidance, order-based electronic alerts recommending alternative culture sites rather than BCx when appropriate (e.g., urine culture for urinary tract infection [UTI]), and soft stop warnings for repeating BCx within 48 hours (Supplemental Materials).

SEP-1 compliance was abstracted per CMS criteria using a random sample of approximately 60 cases per quarter. SEP-1 failures due to BCx collection were analyzed and categorized by timing of antibiotics relative to BCx: short delay (BCx >0–3 hours after antibiotics), long delay (>3 to <24 hours after antibiotics), and no BCx (no BCX within 24 hours after antibiotics). Failures due to no BCx obtained underwent chart review with two physician adjudication (JHR and TVS) to determine infectious source, alternative culture obtainment, clinical adjudication to determine if BCx may have changed management or outcomes, and assignment of pre-test probability of bacteremia (non-infectious causes were assigned 0% probability).^3^ BCx failures were compared descriptively.

## Results

**Figure 1.**
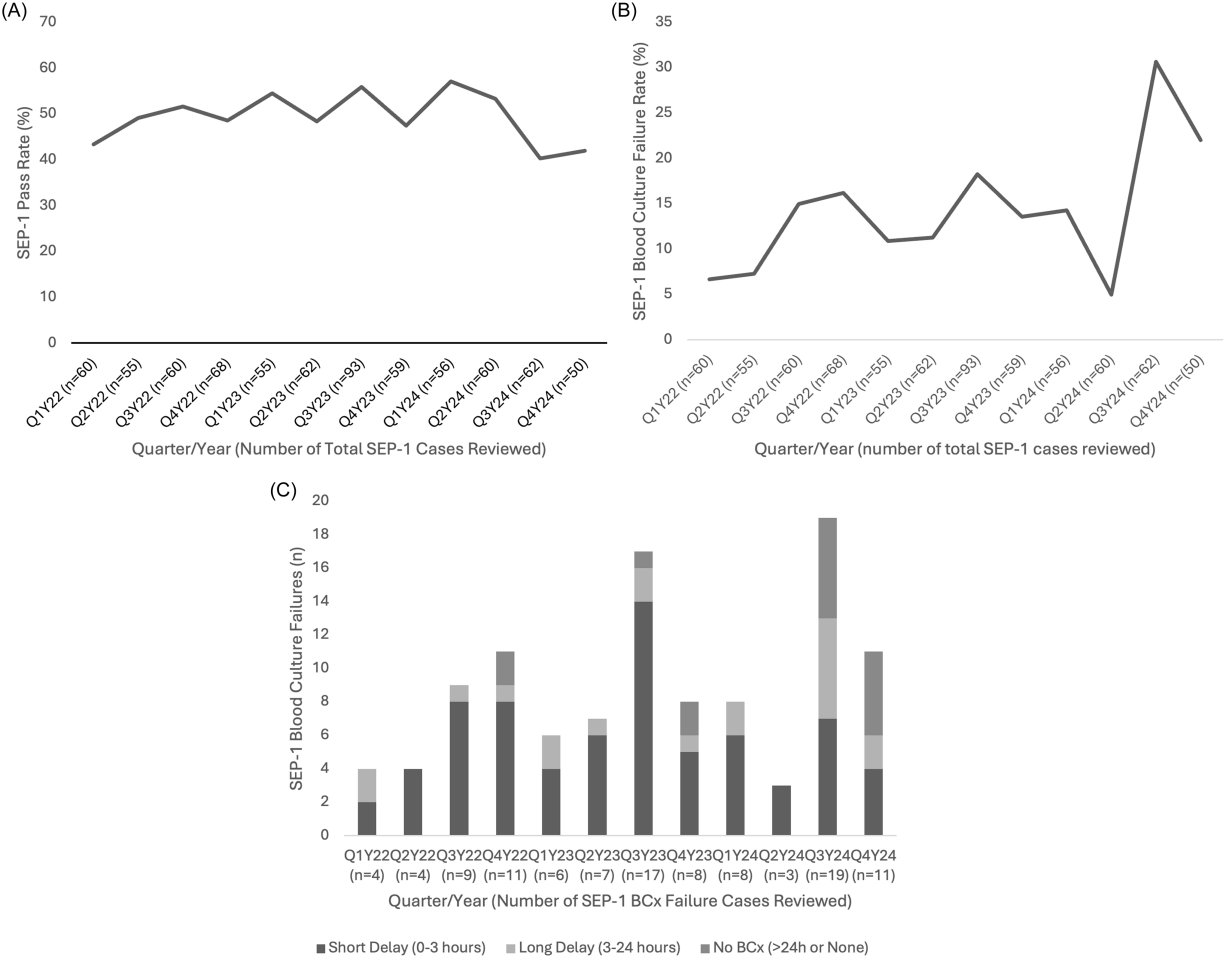
A) SEP-1 Pass Rate by Quarter from 2022 to 2024; B) SEP-1 Failure Rate Related to Blood Cultures by Quarter from 2022 to 2024; C) SEP-1 Blood Culture Failure Reasons by Quarter from 2022 to 2024.

Over 12 quarters, 740 SEP-1 cases were assessed for compliance (mean 61.6 cases/quarter); these were selected from 4728 eligible SEP-1 cases (inpatients age ≥18 with qualifying ICD-10 codes for sepsis), representing 15.7% sampling. Mean SEP-1 success rate pre-shortage was 51.1% (321/628) compared to 41.1% (46/112) during the shortage (Figure 1A). SEP-1 failures due to BCx timing were the reason for SEP-1 failure in 12.3% (77/628) of pre-shortage cases and increased to 26.8% (30/112) during the shortage (Figure 1B). Pre-shortage BCx SEP-1 failures were primarily due to short delays (60/77, 77.9%) with infrequent long delays (12/77, 15.6%) or no BCx (5/77, 6.5%) (Figure 1C). During the shortage, BCx failures were more commonly due to no BCx obtained (11/30, 36.7%) and long delays (8/30, 26.7%).

Among 16 cases without BCx, 12 (75%) occurred during the shortage. All patients with no BCx obtained had clinical evidence of sepsis or septic shock. Upon adjudication, positive BCx would have potentially changed management in only 2/16 (12.5%) cases (Table 1). In one of these cases, BCx were ordered but not obtained. Reasons BCx would not have changed management included UTI or pyelonephritis with positive urine culture (8/16), non-infectious diagnoses or infections not associated with bacteremia (3/16), and community-acquired peritonitis with surgery (3/16). During the BCx shortage, 7/8 UTIs had positive urine cultures, 1 of 2 peritonitis cases had positive intra-abdominal cultures, and 2 cases had non-bacteremic syndromes (viral infection/lymphoma and hypovolemia). Thirteen of sixteen (81.3%) patients survived hospitalization and had no infection-related readmissions or infection recurrences (Table 1).

**Table 1.** Adjudicated SEP-1 case failures due to no BCx obtained

Pre- versus during shortage	Retrospective clinical diagnosis	Alternative culture results	Outcome	Adjudicated Probability of Bacteremia from Fabre et al^3^	Would BCx Change Management and Why?
Pre-Shortage	Pulmonary Edema	RVP negative Urine Antigens for *Streptococcus pneumoniae* and *Legionella* negative	Alive, no recurrent infection	0%	No, non-infectious
Pre-Shortage	UTI	UCx with *Escherichia coli*	Alive, no recurrent infection	20-50%	No, had positive UCx
Pre-Shortage	Cholangitis	None	No in-hospital mortality, but had recurrent infection at 6 weeks, died from complications of non-infectious condition	20–50%	Potentially, BCx are recommended for cholangitis and no alternative cultures were obtained. BCx were ordered, but not obtained.
Pre-Shortage	Peritonitis due to Perforated Viscus	None	Alive, no recurrent infection	11%	Unlikely in setting of community-acquired peritonitis with surgical management
Shortage	UTI	UCx with *E. coli* and *Proteus mirabilis*	Alive, no recurrent infection	20–50%	No, had positive UCx
Shortage	Viral Pneumonia	Sputum culture negative Urine Antigens for *S. pneumoniae* and *Legionella* negative	No in-hospital mortality, but was re-admitted with non-infectious diagnosis and died of complications	0%	No, had viral infection
Shortage	UTI	UCx with *E. coli*	Alive, no recurrent infection	20–50%	No, had positive UCx
Shortage	UTI	UCx with *P. mirabilis*	Alive, no recurrent infection	20–50%	No, had positive UCx
Shortage	UTI	UCx with *E. coli*	Alive, no recurrent infection	20–50%	No, had positive UCx
Shortage	Peritonitis due to Perforated Viscus	Peritoneal fluid with *E. coli*, *Pseudomonas aeruginosa*, *Clostridium tertium*, anaerobes	Alive, no recurrent infection	11%	No, had positive peritoneal culture
Shortage	UTI	UCx with <10K mixed urogenital flora	Alive, no recurrent infection	20–50%	Potentially, UCx were negative and patient had septic shock
Shortage	Peritonitis due to Perforated Viscus	None	Alive, recurrent infection despite effective antibiotic administration	11%	Unlikely in setting of community-acquired peritonitis with surgical management
Shortage	UTI and respiratory tract infection	UCx with *Klebsiella pneumoniae*, RVP with rhinovirus/enterovirus	Alive, no recurrent infection	20–50%	No, had positive UCx
Shortage	UTI	UCx with *E. coli*	Alive, no recurrent infection	20–50%	No, had positive UCx
Shortage	Hypovolemia	RVP negative	Alive, re-hospitalized due to similar episode to initial episode	0%	No, non-infectious
Shortage	UTI, Prostatitis	UCx with *Enterococcus faecalis*	Alive, no recurrent infection	20–50%	No, had positive UCx

RVP, Respiratory Viral Panel; UCx, Urine Culture; UTI, Urinary Tract Infection.

## Discussion

During the BCx shortage, we observed a relative increase in SEP-1 failures, predominantly driven by late or no BCx obtainment. The proportion of SEP-1 failures due to BCx failures (12.3%) pre-shortage compares similarly to a previous cohort (15.1%), and our subsequent increase to >25% of all failures during the shortage is notable.^6^

While the majority of BCx failures pre-shortage were due to patients receiving antibiotics shortly before BCx obtainment, the proportion due to no BCx increased during the shortage. For most patients where BCx were not obtained, it is unlikely positive BCx results would have changed clinical management. Only two patients in our study may have benefited from BCx, with one of them having BCx ordered but were not obtained, suggesting a process failure rather than an issue with BCx stewardship. Situations in which no BCx were obtained during the shortage may have represented conscious decisions by clinicians to conserve BCx supply using evidence-based BCx stewardship guidance, yet they resulted in SEP-1 failures. A multicenter hospital network decreased BCx use by >20% during the shortage without a significant change in sepsis-related mortality, suggesting BCx reduction is safe.^7^

BCx are the gold standard for detecting bloodstream infection and can be invaluable for targeting antibiotics and identifying potential sources of infection, especially in undifferentiated sepsis. However, identification of bloodstream infection does not always result in altered clinical management and even in sepsis may be low yield compared to site-specific cultures. For example, in pyelonephritis, BCx yield is 20–50%, whereas the yield of a urine culture is >95%; concordance between blood and urine cultures exceeds 93%.^3^ Additionally, uncomplicated gram-negative bacteremia and pyelonephritis can both be treated with 7 days of antibiotics with oral transition, so antibiotic management rarely changes even with positive BCx.^8^ Guidelines recommend BCx and intra-abdominal cultures in patients with community-acquired peritonitis related to a perforated viscus with hemodynamic instability and fever.^9^ Peritoneal culture yield is significantly higher than BCx in one study: 69% and 11%, respectively.^10^ In addition, BCx results are usually monomicrobial, while peritoneal culture are more frequently polymicrobial, suggesting BCx are inadequate assessments of the causative pathogens.^11^ Thus, clinicians can effectively treat these syndromes without BCx if alternative cultures are obtained. One concern with not obtaining BCx in sepsis may be if the initial suspected diagnosis is incorrect or if alternative cultures are not obtained in a timely manner. When the diagnosis is apparent, clinicians should be given the opportunity to practice high-value care by conserving resources rather than fear penalization by CMS. If sepsis mimickers can be identified readily, this obviates the need for BCx or antibiotic therapy.^2^

Diagnostic stewardship interventions focus on improving patient outcomes, requiring a balance between decreasing unnecessary use and optimizing yield. Unnecessary BCx are associated with patient harm, waste of resources, and even when positive, do not consistently change management.^3^ However, severely ill patients (e.g., in septic shock) warrant rapid assessment and antibiotic administration. These two competing priorities must be balanced in the creation of metrics such as SEP-1. Unfortunately, the evidence supporting SEP-1 improving outcomes in sepsis without shock is lacking, with IDSA recommending SEP-1 be limited to septic shock alone.^2^ We believe our data similarly supports re-evaluating BCx requirements in SEP-1, as its mandates may limit diagnostic stewardship efforts and clinician autonomy.

Limitations include small sample size of SEP-1 cases from a single hospital system. Formal statistics or trend analyses were not performed due to the sample size and limited shortage duration. Retrospective adjudication of BCx necessity is challenging, as BCx are the only way to define bacteremia. Missing bacteremia may result in shorter than recommended antibiotic durations, which was observed in this cohort. However, the absence of recurrent infections in most patients suggests that bacteremia was either not present or was adequately treated. Analyses of overall effects of the BCx shortage on sepsis-related mortality were not assessed. Further studies of the relationship between BCx stewardship and SEP-1 metrics in a larger cohort would be beneficial.

We observed an increase in SEP-1 failures during the BCx shortage, likely driven by efforts to reduce BCx use. Use of alternative culture sites with higher yield than BCx resulted in SEP-1 failures that did not result in patient harm, a situation that other institutions may experience when implementing similar BCx stewardship recommendations. We believe SEP-1 should be re-evaluated, as BCx are not the only means of diagnosing the microbiologic etiology of infections and are low yield in select patients with sepsis and defined infectious or non-infectious syndromes.
